# Mechanism of mitochondrial permeability transition pore induction and damage in the pancreas: inhibition prevents acute pancreatitis by protecting production of ATP

**DOI:** 10.1136/gutjnl-2014-308553

**Published:** 2015-06-12

**Authors:** Rajarshi Mukherjee, Olga A Mareninova, Irina V Odinokova, Wei Huang, John Murphy, Michael Chvanov, Muhammad A Javed, Li Wen, David M Booth, Matthew C Cane, Muhammad Awais, Bruno Gavillet, Rebecca M Pruss, Sophie Schaller, Jeffery D Molkentin, Alexei V Tepikin, Ole H Petersen, Stephen J Pandol, Ilya Gukovsky, David N Criddle, Anna S Gukovskaya, Robert Sutton

**Affiliations:** 1 NIHR Liverpool Pancreas Biomedical Research Unit, Royal Liverpool University Hospital, Liverpool, UK; 2 Institute of Translational Medicine, University of Liverpool, Liverpool, UK; 3 Veterans Affairs Greater Los Angeles Healthcare System, University of California Los Angeles and Southern California Research Center for Alcoholic Liver and Pancreatic Diseases and Cirrhosis, Los Angeles, California, USA; 4 Institute of Theoretical and Experimental Biophysics, Russian Academy of Sciences, Pushchino, Russia; 5 Department of Integrated Traditional and Western Medicine, Sichuan Provincial Pancreatitis Centre, West China Hospital, Sichuan University, Chengdu, People's Republic of China; 6 Debiopharm Research and Manufacturing S.A., Lausanne, Switzerland; 7 Trophos S.A., Marseille, France; 8 Howard Hughes Medical Institute, Children's Hospital Medical Center, Cincinnati, Ohio, USA; 9 Cardiff School of Biosciences, University of Cardiff, Cardiff, Wales, UK

**Keywords:** ACUTE PANCREATITIS, CELL SIGNALLING, CELL DEATH

## Abstract

**Objective:**

Acute pancreatitis is caused by toxins that induce acinar cell calcium overload, zymogen activation, cytokine release and cell death, yet is without specific drug therapy. Mitochondrial dysfunction has been implicated but the mechanism not established.

**Design:**

We investigated the mechanism of induction and consequences of the mitochondrial permeability transition pore (MPTP) in the pancreas using cell biological methods including confocal microscopy, patch clamp technology and multiple clinically representative disease models. Effects of genetic and pharmacological inhibition of the MPTP were examined in isolated murine and human pancreatic acinar cells, and in hyperstimulation, bile acid, alcoholic and choline-deficient, ethionine-supplemented acute pancreatitis.

**Results:**

MPTP opening was mediated by toxin-induced inositol trisphosphate and ryanodine receptor calcium channel release, and resulted in diminished ATP production, leading to impaired calcium clearance, defective autophagy, zymogen activation, cytokine production, phosphoglycerate mutase 5 activation and necrosis, which was prevented by intracellular ATP supplementation. When MPTP opening was inhibited genetically or pharmacologically, all biochemical, immunological and histopathological responses of acute pancreatitis in all four models were reduced or abolished.

**Conclusions:**

This work demonstrates the mechanism and consequences of MPTP opening to be fundamental to multiple forms of acute pancreatitis and validates the MPTP as a drug target for this disease.

**Significance of this study**

**What is already known on this subject?**

- Toxins that induce acute pancreatitis cause pancreatic acinar cell calcium overload, intracellular zymogen activation, cytokine release and cell death.
- Mitochondrial matrix calcium overload induces opening of the mitochondrial permeability transition pore (MPTP), a non-specific inner mitochondrial membrane channel that causes loss of mitochondrial membrane potential essential to ATP production.
- Calcium-induced opening of the MPTP occurs in acute pancreatitis, but the mechanism and consequences of this process have not been established.

**What are the new findings?**

- Toxins that cause acute pancreatitis induce the MPTP in isolated murine and human pancreatic acinar cells via second messenger receptor calcium channel release and mitochondrial calcium but not reactive oxygen species overload, resulting in mitochondrial depolarisation, impaired ATP production and necrosis.
- Pancreatitis toxin-induced MPTP opening causes activation of phosphoglycerate mutase 5, which executes necrosis, and retarded autophagy, which causes accumulation of activated digestive enzymes.
- Specific genetic or pharmacological inhibition of MPTP opening in a diverse range of clinically relevant mouse models dramatically improves all local pancreatic, systemic and distant pulmonary pathological responses.

**How might it impact on clinical practice in the foreseeable future?**

- The demonstration of identical mechanisms in human as in murine pancreatic acinar cells indicates that the findings that establish MPTP opening to be of critical importance in experimental acute pancreatitis are likely to be of major importance in clinical acute pancreatitis.
- This study has shown the effectiveness in experimental acute pancreatitis of several drugs that target molecules that regulate the MPTP and that could be developed for the treatment of clinical acute pancreatitis.
- Translational drug discovery and development programmes that target the MPTP could provide specific, effective treatments for clinical acute pancreatitis.

## Introduction

Pancreatic necrosis, systemic inflammatory response syndrome, multiple organ failure and sepsis are characteristic of severe acute pancreatitis (AP), which results in death of one in four patients and is without specific drug therapy.^1^ ^2^ As the pancreatic acinar cell is an initial site of injury,^1^ ^3^ commonly initiated by bile or ethanol excess, investigation of its behaviour in response to toxins that induce AP may identify new drug targets. This cell typifies non-excitable exocrine cells with a high secretory turnover heavily dependent on mitochondrial production of ATP.^4^ While zymogen activation has long been considered the principal mechanism of injury,^1^ ^3^ mitochondrial dysfunction has been implicated increasingly,^5–11^ presumed consequent upon intracellular calcium overload induced by toxins that include bile acids and ethanol metabolites.^6^ ^11^ ^12^ Mitochondrial uptake of calcium drives normal cellular bioenergetics, but high calcium loads induce increasingly drastic responses culminating in necrosis.^13^ Mitochondrial matrix calcium overload leads to opening of the mitochondrial permeability transition pore (MPTP), a non-specific channel that forms in the inner mitochondrial membrane allowing passage of particles under 1500 Da, causing loss of mitochondrial membrane potential (Δψ_m_) essential to ATP production;^13^ recent evidence implicates F_0_F_1_ ATP synthase in MPTP formation.^14^ ^15^ MPTP opening is physiological in low conductance mode releasing calcium and reactive oxygen species (ROS) to match metabolism with workload,^16^ but pathological in high conductance mode compromising ATP production and inducing cell death;^13^ both functions are regulated by the mitochondrial matrix protein peptidyl-prolyl *cis-trans* isomerase (PPI, cyclophilin) D (also known as cyclophilin F).^17^

Previous limited studies found that MPTP opening can occur in pancreatitis;^5^ ^9^ ^18^ we found cyclophilin D knockout to ameliorate AP induced by ethanol and cyclosporine,^9^ but in a model with no clinical correlate. How the MPTP is induced in pancreatic acinar cells has not been determined, nor what role intracellular calcium might play and whether there are downstream consequences in AP. Therefore, we sought to undertake a novel, wide ranging and detailed study to determine the mechanism and significance of MPTP opening in AP.

We report that MPTP opening is critical to all forms of pancreatitis investigated, causing diminished ATP production, defective autophagy, zymogen activation, cytokine release, phosphoglycerate mutase family member 5 (PGAM5) activation^19^ and necrosis. Pharmacological or genetic MPTP inhibition in murine or human pancreatic acinar cells protected Δψ_m_, ATP production, autophagy and prevented necrosis from pancreatitis toxin-induced calcium release via inositol trisphosphate and ryanodine (IP_3_R, RyR) calcium channels. This mechanism was confirmed consistently across four dissimilar, clinically relevant, in vivo models of AP. All characteristic local and systemic pathological responses were greatly reduced or abolished in cyclophilin D knockout mice (*Ppif^−/−^*)^20^ and wild type (Wt) mice treated with MPTP inhibitors, confirming that MPTP opening is a fundamental pathological mechanism in AP.

## Methods

### Animals

Cyclophilin D-deficient mice were generated by targeted disruption of the *Ppif* gene^20^ and provided by Dr Derek Yellon (University College London, UK) and Dr Michael A Forte (Oregon Health and Sciences University, USA). Transgenic green fluorescent protein (GFP)-LC3 mice^21^ were a gift from Dr N Mizushima (Tokyo Medical and Dental University and RIKEN BioResourse Center, Japan). All experiments comparing Wt and *Ppif^−/−^* were conducted using C57BL/6 mice; experiments using toxins on Wt cells alone used CD1 mice.

### Preparation of isolated pancreatic acinar cells and mitochondria

Normal human pancreata samples (∼1 cm×1 cm×1 mm, not devascularised during surgery before removal) were placed in a solution of (mM): 140 NaCl, 4.7 KCl, 1.13 MgCl_2_, 1 CaCl_2_, 10 D-glucose, 10 HEPES (adjusted to pH 7.35 using NaOH) at 4°C; sampling to start of cell isolation (or slicing below) was <10 min in every case. All experiments were at room temperature (23–25°C, except where stated) and cells used within 4 h of isolation. Isolation of murine^7^ and human^22^ pancreatic acinar cells was as described. Isolated murine cells were incubated at 37°C in 199 medium with or without 10 nM cholecystokinin-8 (CCK-8) or 500 µM taurolithocholic acid sulfate (TLCS); drug pretreatment was applied for 30 min. Mitochondria were isolated from mouse pancreata as described.^23^

### Confocal fluorescence microscopy

Cells and tissue were viewed using Zeiss LSM510 and LSM710 systems (Carl Zeiss Jena GmbH), typically with a 63x C-Apochromat water immersion objective (aperture at 1.2) after loading with Fluo-4 (3 µM; excitation 488 nm, emission 505 nm) and tetramethyl rhodamine methyl ester (50 nM; excitation 543 nm, emission >550 nm) to assess cytosolic calcium and mitochondrial membrane potential, with simultaneous measurements of NAD(P)H autofluorescence (excitation 351 nm, emission 385–470 nm) to assess mitochondrial metabolism. The protonophore carbonyl cyanide *m*-chlorophenyl hydrazone (CCCP) was applied to dissipate Δψ_m_ as a positive control. ROS were assessed after loading with 5-chloromethyl-2,7-dichlorodihydrofluorescein diacetate acetyl ester (4.5 μM; excitation 488 nm, emission 505–550 nm) for 10 min at 37°C.^12^ R110-aspartic acid amide (20 μM; excitation 488 nm, emission >505 nm) and propidium iodide (PI 1 µM; excitation 488 nm, emission 630–693 nm) were used to assess general caspase activation and plasma membrane rupture. Thirty random fields of view were taken of each isolate and the percentage number of cells displaying caspase activity or PI uptake counted per field, averaged across fields as mean±SEM (minimum three mice/group). PI was used in patched cells (below), as was Mg Green (4 µM, excitation 476 nm, emission 500–550 nm), to monitor intracellular ATP concentrations.^6^ Murine pancreas lobules were incubated with/without 500 μM TLCS and stained with Sytox Orange^24^ (500 nM, excitation 543 nm, emission >560 nm), which like PI only stains cells with ruptured cell membranes: uptake was determined every two hours by % area tissue stained.

### Patch-clamp current recording

The whole-cell configuration was used to record ICl_Ca_ from single cells while recording cytosolic calcium (Fluo-4).^25^ Patch-pipettes were pulled from borosilicate glass capillaries (Harvard Apparatus) with resistance of 2–3 MΩ when filled with a solution of (mM): 140 KCl; 1.5 MgCl_2_; 2 MgATP; 10 HEPES; 0.1 ethylene glycol tetraacetic acid, pH 7.2. The second messenger IP_3_, cyclic ADP ribose (ADPR) or nicotinic acid adenine dinucleotide phosphate (NAADP) was added to the pipette solution at quasi-physiological concentrations (1–10 μM); in some experiments, MgATP was omitted, while in others MgATP was raised to 4 mM. Patched cells were exposed to TLCS (10 µM with second messenger or ACh or CCK-8, otherwise 200 µM) or palmitoleic acid ethyl ester (POAEE, 10 µM) for 20 min then stained with PI. Whole-cell currents were sampled at 10 kHz (EPS8 amplifier and Pulse software, HEKA) from a holding potential of −30 mV, with steps to +10 mV, beyond the reversal potential (0 mV) of the two Ca^2+^-dependent currents through Cl^−^ and non-selective cationic channels.^25^

### Experimental AP

Caerulein (CER)-AP was induced in male (25–30 g) mice by 7 hourly intraperitoneal injections of 50 μg/kg CER;^26^ controls received saline; sacrifice was made 7 h after the first injection for *Ppif^−/−^* comparisons with Wt, or 12 h for assessment of DEB025 or TRO40303 in Wt. Dosing was determined by prior pharmacokinetic and pharmacodynamic studies (data not shown), which identified optimum regimens of 12 hourly injections of DEB025 at 10 mg/kg or TRO40303 at 3 mg/kg. TLCS-AP was induced as described^27^ by retrograde injection of the pancreatic duct with 3 mM TLCS while controls had ductal injection of saline; sacrifice was 24 h later. Fatty acid ethyl ester (FAEE)-AP was induced by 2 hourly intraperitoneal 1.35 g/kg ethanol and 150 mg/kg palmitoleic acid (POA)^11^ with controls receiving saline; sacrifice was 24 h later. Choline-deficient ethionine-supplemented (CDE)-AP was induced in young female mice (14–15 g) fasted overnight and fed CDE^28^ or regular chow for 48 h, then sacrificed.

### Histopathology

Pancreatic necrosis was measured on H&E sections as described and apoptosis with TUNEL.^7^ Two independent, blinded investigators scored oedema, leucocyte infiltration and necrosis (0–3) on ×10 high-power fields/slide/mouse. Scores were summated (mean±SEM ≥6 mice/group; ≥4 for CDE-AP).

### Statistical analysis

Data are presented as mean±SEM. Analysis was by two-tailed Student's t test or χ^2^ test, with p values <0.05 considered significant**.**

### Study approval

For preparation of pancreas tissue slices and lobules, measurement of isolated mitochondrial responses, electron microscopy, immunofluorescence, further assessment of disease parameters in experimental AP and details of chemicals and reagents, see online supplementary materials.

## Results

### Pharmacological MPTP inhibition prevents pancreatitis toxin-induced mitochondrial impairment and necrotic cell death pathway activation

**Figure 1:**
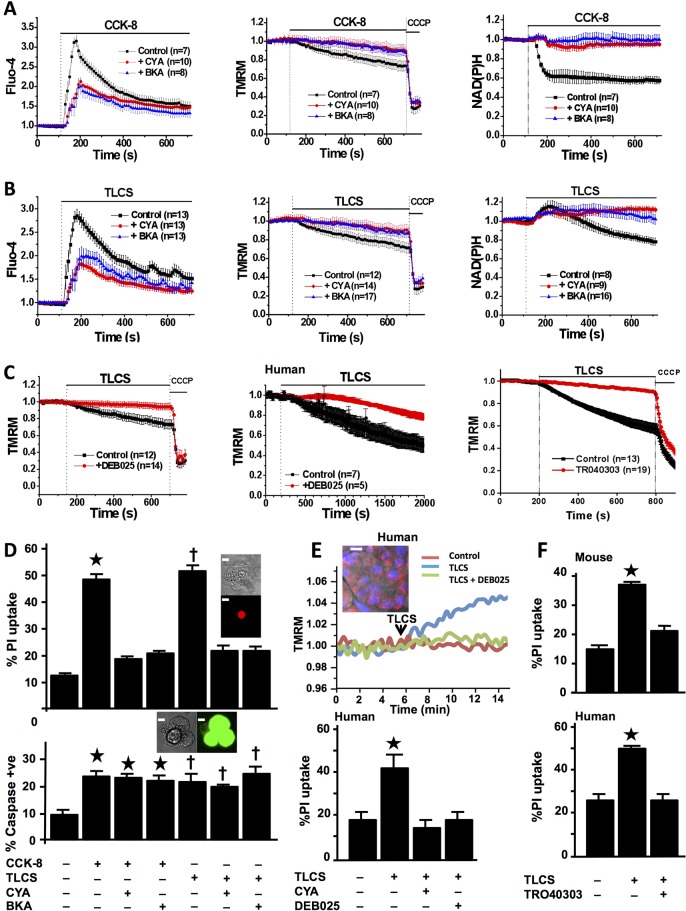
Mitochondrial permeability transition pore (MPTP) inhibitors prevent mitochondrial impairment and necrosis of freshly isolated murine and human pancreatic acinar cells (confocal fluorescence; mean±SEM ratio to basal, F/F_0_; n=no. of experiments). (A) Cholecystokinin-8 (CCK-8) (10 nM) induced large cytosolic calcium elevations (Fluo-4, left), falls in Δψ_m_ (tetramethyl rhodamine methyl ester, TMRM; positive control, protonophore carbonyl cyanide *m*-chloro phenyl hydrazone, CCCP, middle) and NAD(P)H autofluorescence (right), showing protection of Δψ_m_ and NAD(P)H by cyclosporin A (CYA, 5 µM) or bongkrekic acid (BKA, 50 µM) (pretreatment for 30 min at room temperature during loading of fluorescent dyes). (B) Taurolithocholic acid sulfate (TLCS) (500 µM) induced similar changes in calcium, Δψ_m_ and NAD(P)H, with similar protection by CYA and BKA. (C) Protection of Δψ_m_ from TLCS (500 µM) by pretreatment with DEB025 (100 nM) in murine (left) and human (middle) pancreatic acinar cells, and with TRO40303 (10 μM, right) in murine cells. (D) CYA or BKA protected cells from early plasma membrane rupture (top, % cells showing propidium iodide (PI) uptake as inset; *p<0.05 CCK-8 vs control or with inhibitor; ^†^p<0.05 TLCS vs control or with inhibitor) but not from caspase activation (bottom, % cells showing general caspase substrate fluorescence as inset; *p<0.05, control vs all CCK-8 groups; ^†^p<0.05 control vs all TLCS groups; white bars=5 μm). (E) Typical rise in TMRM dequench fluorescence^31^ emitted by normal fresh human pancreatic tissue slice in response to TLCS (500 μM) and protection by DEB025 (100 nM; upper panel; inset shows confocal image of human pancreatic tissue slice (mitochondrial (TMRM, red) and nuclear (Hoescht, blue) fluorescent dyes, white bar=15 μm). Lower panel shows protection from TLCS-induced PI uptake in human pancreatic acinar cells by CYA (100 nM) or DEB025 (100 nM, bottom) and (F) murine (top) and human (bottom) cells by TRO40303 (10 μM) (*p<0.05 TLCS vs control or with CYA, DEB025 or TRO40303).

First, we tested the effect of known MPTP inhibitors on toxin-induced changes in pancreatic acinar cells using cyclosporin A (CYA), which binds to and inhibits cyclophilin D, or bongkrekic acid (BKA), which favours the closed conformation of adenine nucleotide translocase.^29^ We used murine cells hyperstimulated with CCK-8^18^ ^30^ to induce cytosolic and mitochondrial calcium overload,^6^ ^12^ and found loss of Δψ_m_^7^ ^18^ causing decreases in NAD(P)H (figure 1A), reflecting declining ATP production.^4^ The bile acid TLCS^31^ induced similar changes (figure 1B). Both CYA and BKA prevented losses of Δψ_m_ and NAD(P)H. TLCS-induced mitochondrial impairment was completely prevented by the calcium chelator 1,2-bis(o-aminophenoxy)ethane-N,N,N′,N′-tetraacetic acid and was dose dependent (see online supplementary figure S1). We then tested D-MeAla^3^-EtVal^4^-cyclosporine (Alisporivir, DEB025), which inhibits cyclophilin D but is not immunosuppressive^29^ and 3,5-Seco-4-nor-cholestan-5-one oxime-3-ol (TRO40303), which also inhibits MPTP opening;^32^ both prevented decreases of Δψ_m_ in murine and freshly isolated human pancreatic acinar cells (figure 1C). Marked cell death pathway activation was induced by CCK-8 and TLCS; whereas caspase activation occurred in the presence of CYA or BKA, PI uptake was largely prevented (figure 1D). Marked protection from TLCS in human pancreatic acinar cells^22^ and human pancreas slices followed pretreatment with CYA, DEB025 and TRO40303 (figure 1E, F).

### Genetic MPTP inhibition prevents pancreatitis toxin-induced mitochondrial impairment and necrotic cell death pathway activation

**Figure 2:**
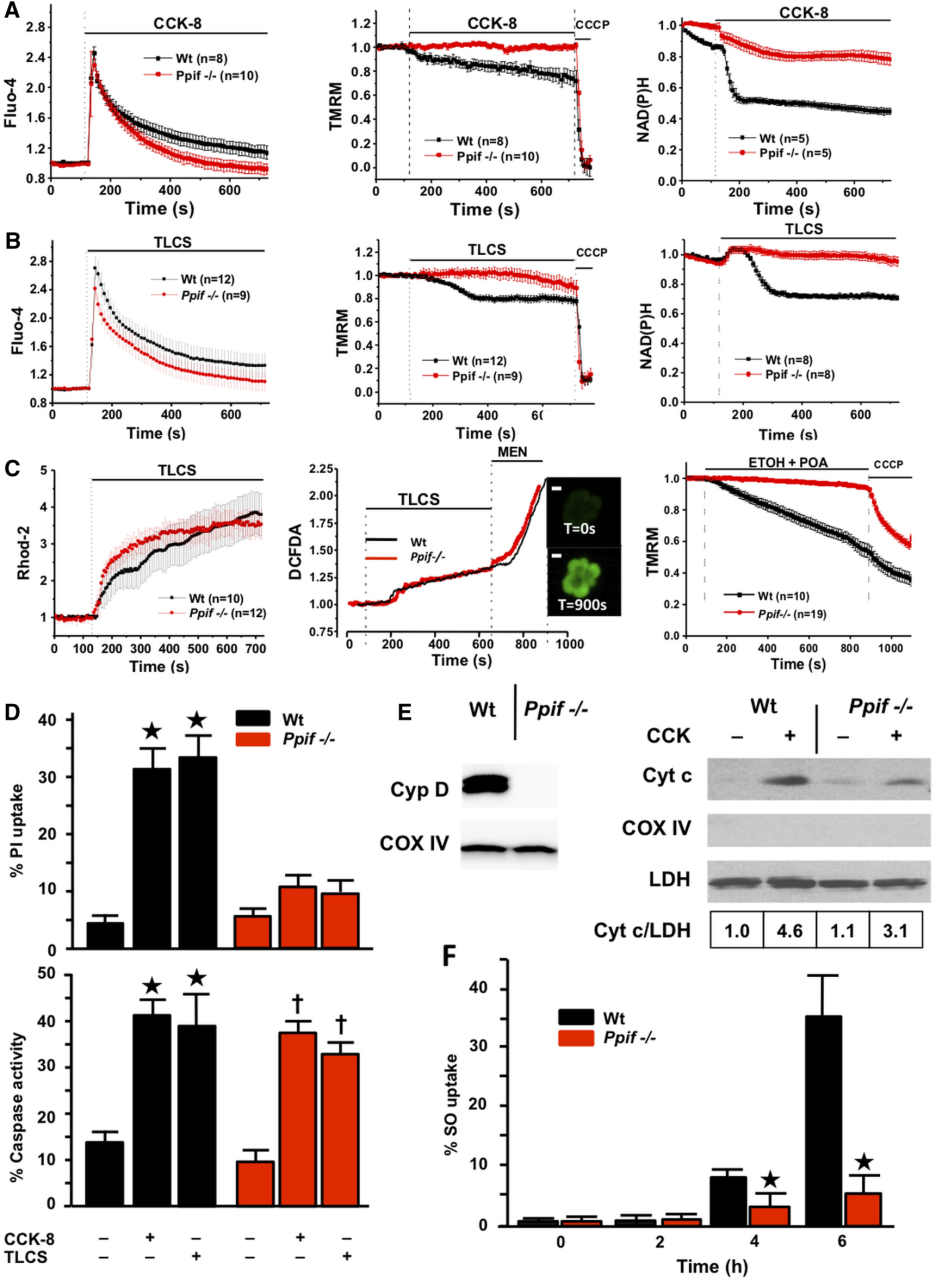
Genetic ablation of cyclophilin D (*Ppif^−/−^*) protects pancreatic acinar cells from pancreatitis toxins (fluorescence mean±SEM, F/F_0_). (A) Cholecystokinin-8 (CCK-8) (10 nM) induced cytosolic calcium elevations (Fluo-4, left) in Wt (C57BL/6) and *Ppif^−/−^* cells, with faster clearance in *Ppif^−/−^*; Δψ_m_ (TMRM, middle) and NAD(P)H (right) were preserved in *Ppif^−/−^* not wild type (Wt) cells. (B) Taurolithocholic acid sulfate (TLCS) (500 µM) induced similar calcium changes, clearing faster in *Ppif^−/−^*; whereas Δψ_m_ and NAD(P)H were preserved in *Ppif^−/−^* not Wt. (C) TLCS (500 μM) induced similar mitochondrial calcium elevations (Rhod-2, left) in *Ppif^−/−^* and Wt cells, as well as similar reactive oxygen species (ROS) elevations (DCFDA, middle) in *Ppif^−/−^* and Wt cells (menadione, MEN oxidant control); insets show ROS-sensitive DCFDA cell fluorescence (white bars=10 μm); ethanol (ETOH, 10 mM) and palmitoleic acid (POA, 20 μM) induced falls of Δψ_m_ (right) in Wt not *Ppif^−/−^* cells. (D) Significantly increased propidium iodide (PI) uptake in Wt not *Ppif^−/−^* cells after CCK-8 (10 nM) or TLCS (500 µM) (top, *p<0.05 toxin in Wt versus no toxin or toxin in *Ppif^−/−^*), but similar general caspase activation (bottom, *p<0.05 no toxin vs each toxin group). (E) Cyclophilin absence in *Ppif^−/−^* pancreas (immunoblot, left) and cytochrome *c* (Cyt c) cytosolic fraction immunoblots (densitometry normalised to lactate dehydrogenase (LDH), Cox IV to rule out mitochondrial contamination, right) showed Cyt c release after CCK-8 by Wt and less by *Ppif^−/−^* mitochondria. (F) Necrotic cell death pathway activation (Sytox Orange; SO) from TLCS (500 µM) was delayed in *Ppif^−/−^* vs Wt pancreas lobules (*p<0.05).

Cytosolic calcium changes were significantly less marked in pharmacologically treated than control cells (seen with CYA and BKA due to an initial release of calcium from cell stores, but not with DEB025 or TRO40303, see online supplementary figure S1), which might reduce mitochondrial calcium loading, so we examined effects of genetic deletion (*Ppif^−/−^*) of cyclophilin D.^20^ Comparison of *Ppif^−/−^* and Wt (C57BL/6) cells showed CCK-8-induced cytosolic calcium elevations were similar, but in *Ppif^−/−^* calcium clearance was significantly faster (mean±SE area under curve (F/F_0_×s) 199.61±26.45 vs 262.35±30.73 in Wt, p<0.05), a function of calcium ATPase pumps (figure 2A, left). In Wt cells, Δψ_m_ and NAD(P)H fell steadily, but not in *Ppif^−/−^* (figure 2A, middle and right). TLCS induced similar changes (figure 2B) with significantly faster calcium clearance in *Ppif^−/−^* cells (243.82±32.34 vs 378.92±45.98 in Wt, p<0.05), despite little difference detected in mitochondrial calcium loading (figure 2C, left). Subsequent experiments demonstrated no difference between *Ppif^−/−^* and Wt cells in store-operated calcium entry or plasma membrane ATPase calcium pump extrusion (see online supplementary figure S1), consistent with more effective ATP supply in *Ppif^−/−^* compared with Wt cells subjected to CCK-8- or TLCS-induced calcium overload. As TLCS-induced ROS increases promote apoptosis not necrosis of pancreatic acinar cells,^12^ ^23^ we tested whether ROS increases are greater in *Ppif^−/−^* cells and found no differences from Wt (figure 2C, middle), ruling this out as a protective mechanism. Ethanol and POA, which form the toxic FAEE POAEE that induces AP,^11^ also caused marked falls of Δψ_m_ in Wt not *Ppif^−/−^* cells (figure 2C, right). There were marked effects of *Ppif^−/−^* on PI uptake but little on general caspase activation (figure 2D), consistent with a minor role for MPTP opening in pancreatic acinar cell apoptosis.^7^ ^23^ In keeping, cytosolic cytochrome *c* release was seen in both *Ppif^−/−^* and Wt cells after hyperstimulation, although less in *Ppif^−/−^* cells (figure 2E). We also tested pancreatic lobules, more closely representing events in vivo, and found necrotic pathway activation (Sytox Orange uptake)^24^ markedly inhibited in *Ppif^−/−^* (figure 2F).

### Pancreatitis toxin-induced acinar cell MPTP opening causes collapse of ATP production and necrotic cell death pathway activation via second messenger receptor calcium channel release

**Figure 3:**
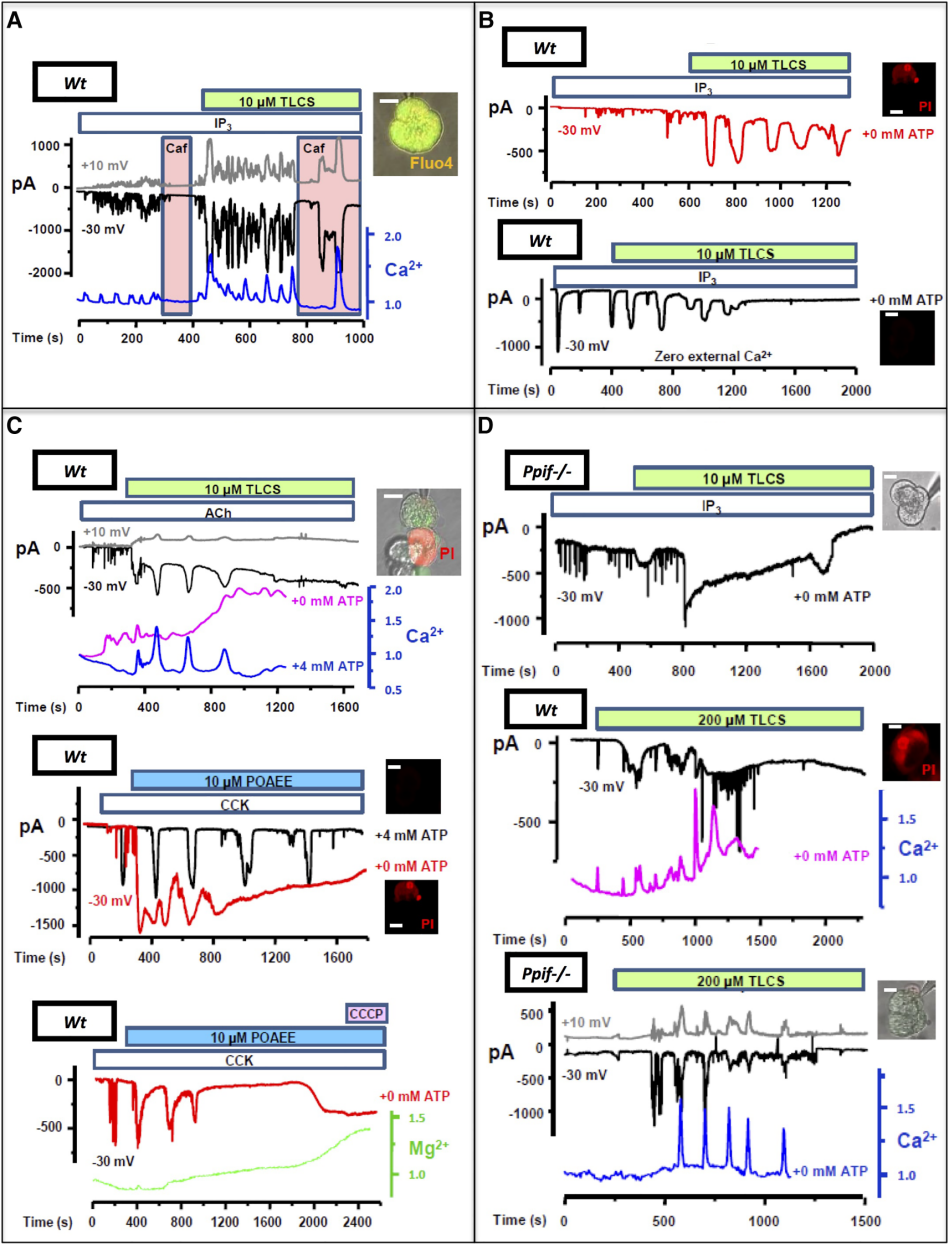
Pancreatitis toxins accelerate calcium release via second messenger receptors causing collapse of ATP production in wild type (Wt) not *Ppif^−/−^* cells (insets, representative cells, green Fluo-4 and/or red propidium iodide (PI) fluorescence, white bars=10 μm). (A) Typical calcium spikes (Fluo-4, F/F_0_, blue) elicited by patched IP_3_ (1–10 µM) were transformed into global, prolonged elevations upon Taurolithocholic acid sulfate (TLCS) (10 µM) application, matched by ICl_Ca_ and non-specific cation currents (−30 mV, black and +10 mV, grey; inset patched cell top), inhibited by caffeine (Caf, pink); (B) top plot: toxic transformation (ICl_Ca_ red, no caffeine) showing PI uptake; bottom plot: without external calcium, transformed signals decreased then disappeared (ICI_Ca_ black, no PI uptake). (C) Top plot: toxic transformation of acetylcholine (Ach) (20 nM) signals by TLCS (10 µM), reduced by pipette ATP (4 mM) preventing PI uptake in patched (ICl_Ca_ and blue calcium trace) but not adjacent (purple calcium trace) cell; middle plot: toxic transformation of cholecystokinin-8 (CCK-8) (1–5 pM) signals by palmitoleic acid ethyl ester (POAEE, 10 μM) (red ICl_Ca_) caused PI uptake, prevented by pipette ATP (black ICl_Ca_, two recordings superimposed); bottom plot: ATP decline (Mg Green; rise indicates increased ADP:ATP ratio) following toxic transformation (3 pM CCK-8 with 10 µM POAEE; carbonyl cyanide m-chlorophenyl hydrazone induced no further ATP decline). (D) Top plot: representative trace showing transformation of IP_3_- (1–10 µM) elicited signal by TLCS (10 µM) did not induce PI uptake in *Ppif^−/−^* cells, without supplementary ATP; middle plot: TLCS (200 μM) alone induced PI uptake in Wt; bottom plot: TLCS (200 μM) did not induce PI uptake in *Ppif^−/−^* cells.

As bile acids and FAEEs induce global, prolonged acinar cytosolic calcium release via IP_3_R and RyR calcium channels,^6^ ^33^ which causes zymogen activation^34^ ^35^ dependent on sustained calcium entry,^36^ we sought to determine how toxin-induced calcium release causes mitochondrial injury and pancreatic acinar cell death. Using patch clamp technology and confocal microscopy, we observed typical apical stimulus-secretion coupling calcium signals elicited by IP_3_ (1–10 μM), matched by calcium-activated Cl^−^ currents^4^ ^25^ (ICl_Ca_). These signals were promptly transformed into global, prolonged (>30 s) cytosolic calcium elevations by low concentrations of TLCS (10 μM, 31 of 33 cells, figure 3A, B) or POAEE (10 μM, 34 of 37 cells, see online supplementary figure S2), followed by PI uptake in Wt cells (figure 3B and see online supplementary table). Application of the non-specific IP_3_R antagonist caffeine^6^ inhibited calcium changes and ICI_Ca_ from both toxins, preventing PI uptake (22 of 22 cells, figure 3A, see online supplementary figure S2 and table), demonstrating dependence of toxic transformation on IP_3_Rs. Necrotic cell death pathway activation was entirely dependent on calcium influx (figure 3B and see online supplementary figure S2). Typical calcium signals and ICI_Ca_ elicited by the RyR ligand cyclic ADPR (cyclic ADPR, 10 μM)^25^ were transformed by TLCS (10 μM), not POAEE; those elicited by NAADP (100 nM)^25^ were transformed by POAEE (10 μM), not TLCS (see online supplementary figure S2). To model events in vivo, quasi-physiological concentrations of CCK-8 or acetylcholine (ACh) were tested with both toxins, again resulting in toxic transformation (24 of 24 cells, no patch pipette, figure 3C, see online supplementary figure S2 and table). Without any second messenger or secretagogue, higher toxin concentrations (TLCS, 200 μM, figure 3D; POAEE 100 μM, data not shown; both inhibited by caffeine) were required to induce global, prolonged calcium elevations. All protocols that induced such elevations sustained by external calcium entry resulted in PI uptake in Wt cells (56 of 60 cells, ≥5 cells with each protocol; figure 3B–D); patched ATP resulted in more efficient calcium clearance and prevented all PI uptake (46 of 46 Wt cells, ≥4 cells with each protocol; p<0.0001), and ATP depletion from toxic transformation without patched ATP was confirmed using Mg Green (figure 3C, see online supplementary figure S2 and table). In all *Ppif^−/−^* cells, there was significantly more efficient calcium clearance, reduced ICI_Ca_ and return to baseline levels with no PI uptake, despite no patched ATP (IP_3_ and TLCS, 10 μM, 17 of 17 cells; TLCS, 200 μM, 7 of 7 cells, figure 3D). These findings identify a primary role for second messenger calcium channel release in MPTP opening induced by pancreatitis toxins, resulting in declining ATP production and necrosis.

### The MPTP determines the sensitivity of pancreatic mitochondria to calcium overload and PGAM5 induction

**Figure 4:**
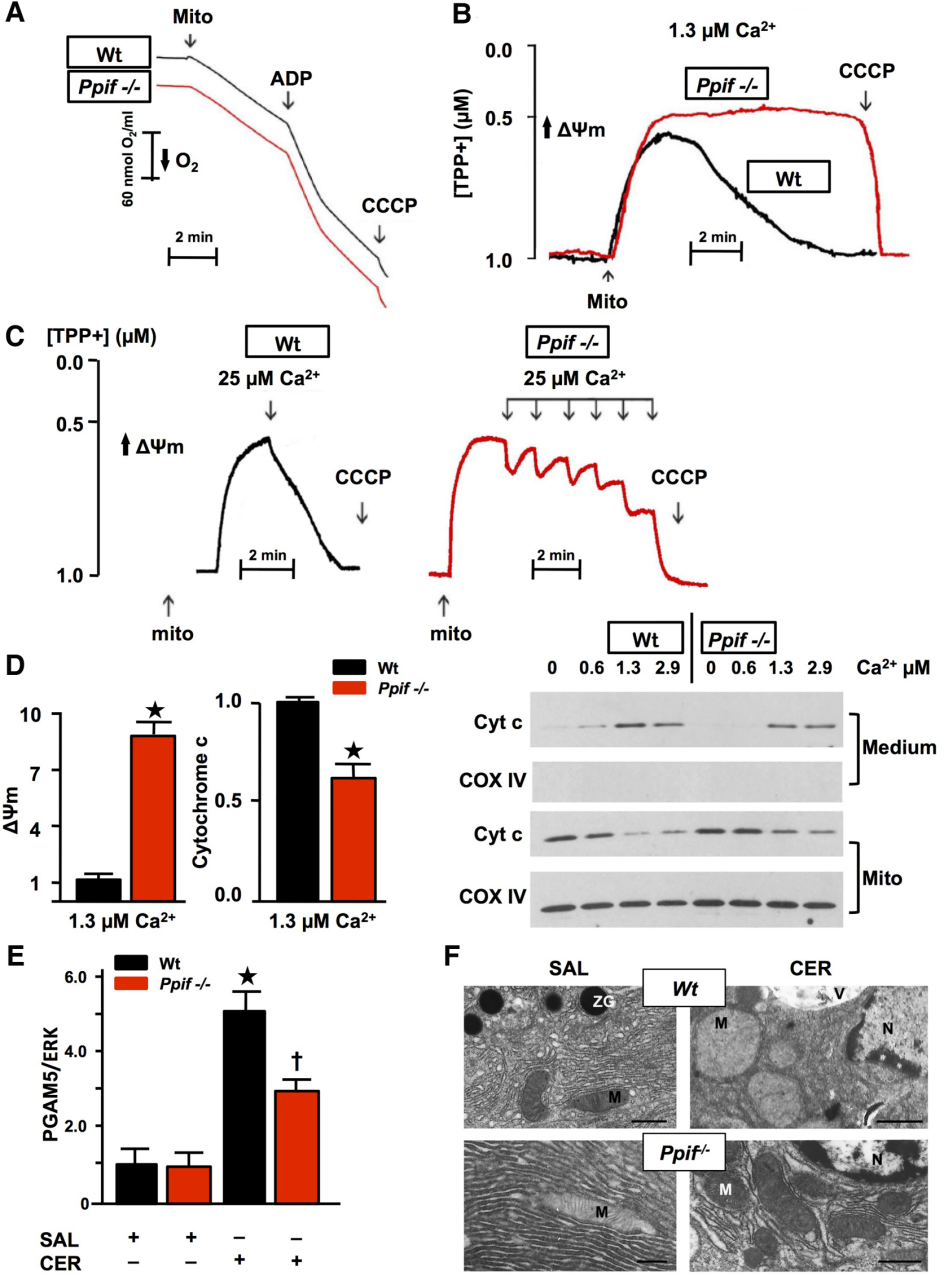
Genetic mitochondrial permeability transition pore (MPTP) inhibition confers resistance of pancreatic mitochondria to calcium-induced loss of Δψ_m_ and PGAM5 induction. (A) Representative Clark-type electrode measurement of oxygen consumption showed no difference between wild type (Wt) and *Ppif^−/−^* mitochondria (Mito; succinate=10 mM, ADP=200 μM, carbonyl cyanide m-chlorophenyl hydrazone (CCCP)=2 μM). (B) Typical TPP^+^-selective electrode measurement of Δψ_m_ with succinate (10 mM) in free ionised calcium clamped at 1.3 µM (calcium/ethylene glycol tetraacetic acid buffers) for 10 min and (C) during pulses of calcium (25 µM), showing resistance of *Ppif^−/−^* mitochondria to loss of Δψ_m_. (D) Δψ_m_ (TPP^+^-selective electrode, left) and cytochrome *c* (Cyt c; densitometry from Medium immunoblot, right) in the same preparations, normalised to Wt. *Ppif^−/−^* pancreatic mitochondria release Cyt c but less than Wt (*p<0.05, means±SEM from >3 preparations), as shown in representative Cyt c immunoblot of medium and mitochondrial pellet (Mito, Cox IV confirmed separation and equal protein loading). (E) Increase in PGAM5 in Wt caerulein acute pancreatitis (CER-AP) pancreata was significantly reduced in *Ppif^−/−^* with representative immunoblot (re-probed for ERK1/2 to confirm equal loading; each lane from an individual animal; 4–6 mice per group; densitometry of PGAM5 as ratio of band intensities to ERK in each sample normalised to saline-treated Wt controls, means±SEM; *p<0.01 CER-AP in Wt vs Wt controls, †p<0.05 CER-AP in *Ppif^−/−^* vs CER-AP in Wt). (F) Electron micrographs of pancreata showing Wt and *Ppif^−/−^* pancreatic acinar cells after induction of CER-AP compared with saline (SAL) controls. Wt pancreatic acinar mitochondria are markedly swollen with loss of cristae in CER-AP compared with normal morphology of *Ppif^−/−^* pancreatic acinar mitochondria in CER-AP and in both Wt and *Ppif^−/−^* saline controls (M, mitochondrion; N, nucleus; V, vacuole; ZG, zymogen granule; black bars 1 μm except top right, 2.5 μm).

To confirm the role of calcium overload in pancreatic acinar cell MPTP opening, we examined responses of isolated *Ppif^−/−^* and Wt pancreatic mitochondria to external calcium. *Ppif^−/−^* and Wt pancreatic mitochondria demonstrated similar capacity to generate ATP, as measured by respiration rate in response to ADP (respiratory control ratio) >3 in the presence of succinate^23^ (figure 4A). Both types of mitochondria maintained Δψ_m_ in zero or 0.6 μM clamped, free ionised calcium for 10 min; in 1.3 µM calcium Wt Δψ_m_ collapsed, whereas *Ppif^−/−^* Δψ_m_ was maintained. While Wt Δψ_m_ was lost after one addition of 25 µM CaCl_2_, *Ppif^−/−^* Δψ_m_ was lost after five successive additions (figure 4B, C). *Ppif^−/−^* pancreatic mitochondria released only 35% less cytochrome *c* than Wt in 1.3 µM calcium (figure 4D), consistent with a modest contribution from MPTP opening to cytochrome c release. To further assess the significance of MPTP opening and falls in Δψ_m_, we measured levels of PGAM5, a mitochondrial executor of necrosis.^19^ Falls in Δψ_m_ cause PGAM5 cleavage from the inner mitochondrial membrane,^37^ and increases in PGAM5 promote necrosis, facilitating mitochondrial fission.^19^ After induction of CER-AP, PGAM5 was increased in Wt but significantly less in *Ppif^−/−^* pancreata (figure 4E), indicating a mitochondrial mechanism for necrosis induced by calcium overload in AP. These changes were associated with marked ballooning of and loss of cristae in Wt but not *Ppif^−/−^* pancreatic acinar mitochondria in CER-AP (figure 4F).

### The MPTP mediates zymogen activation through impaired autophagy

**Figure 5:**
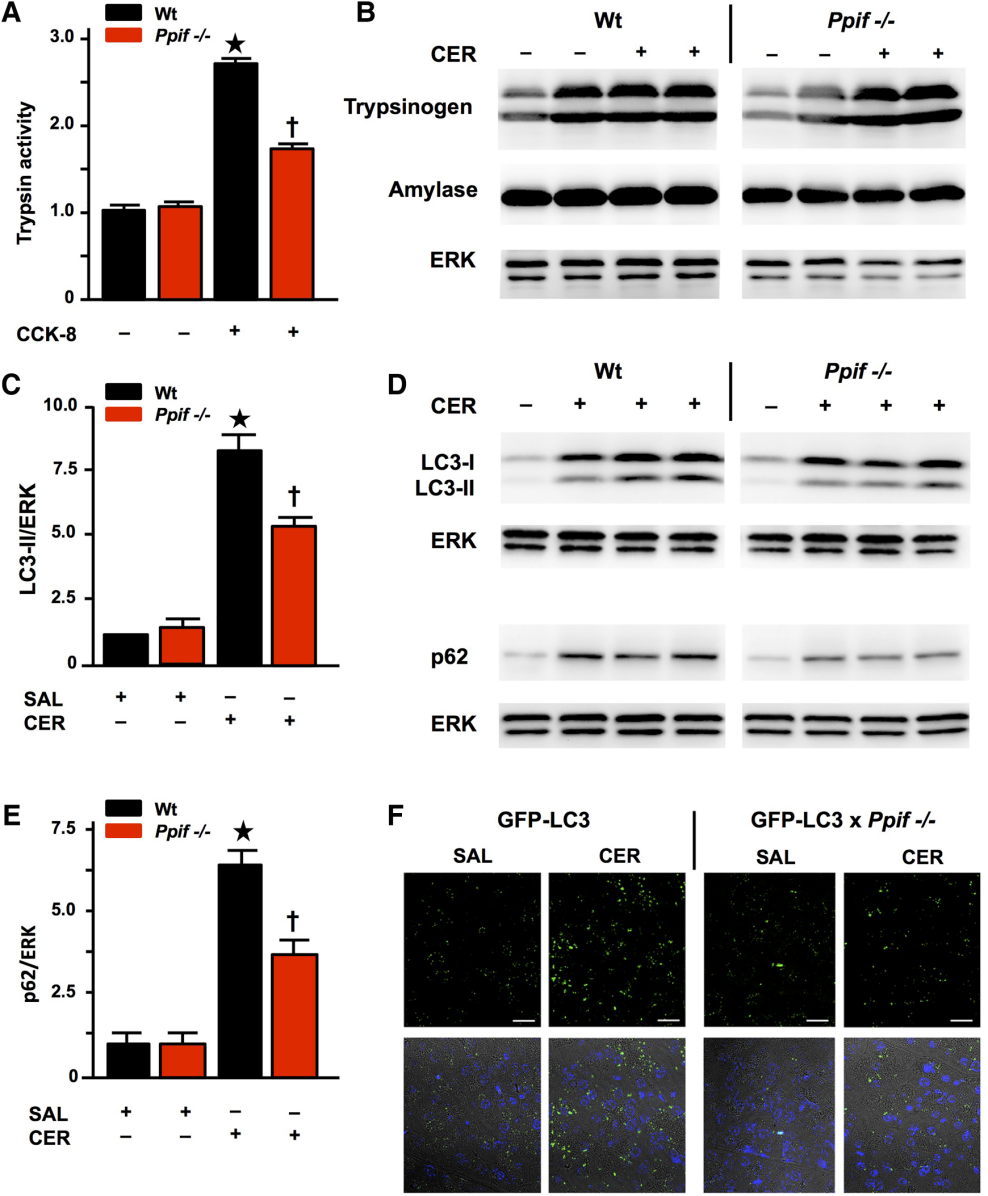
Pancreatitis autophagy impairment and trypsinogen activation are attenuated by genetic mitochondrial permeability transition pore (MPTP) inhibition (*Ppif^−/−^*). (A) Trypsin activity (normalised) following cholecystokinin-8 (CCK-8) (10 nM) hyperstimulation inhibited in *Ppif^−/−^* vs wilt-type (Wt) cells (mean±SEM 6 cell preparations; *p<0.05 Wt CCK-8 vs Wt controls; ^†^p<0.05 *Ppif^−/−^* CCK-8 vs Wt CCK-8). (B) Trypsinogen and amylase content similar in unstimulated and caerulein acute pancreatitis (CER-AP) Wt vs *Ppif^−/−^* pancreata (immunoblot; re-probed for ERK1/2 to confirm equal loading; each lane from an individual animal). (C) Densitometry of LC3-II with (D) representative immunoblots of LC3-II and p62 with (E) densitometry of p62 showing increased levels of these proteins in Wt CER-AP that were both significantly attenuated in *Ppif^−/−^* mice, indicating more efficient autophagic flux (immunoblots re-probed for ERK1/2 to confirm equal loading; each lane from an individual animal; 4–6 mice per group; densitometry of LC3-II or p62 as ratios of band intensities to ERK in each sample normalised to saline-treated Wt controls, means±SEM; *p<0.01 CER-AP in Wt vs Wt controls, ^†^p<0.05 CER-AP in *Ppif^−/−^* vs CER-AP in Wt). (F) Representative images showing attenuation of CER-AP-induced increases in LC3-II puncta by genetic MPTP inhibition in green fluorescent protein (GFP)-LC3×*Ppif^−/−^* compared with GFP-LC3 transgenic mice (upper panels show LC3 puncta in saline-treated pancreas and CER-AP for both strains; lower panels show addition of nuclear stain 4',6-diamidino-2-phenylindole (DAPI)).

Since zymogen activation is considered essential to AP and relates to disease severity,^1^ ^38–40^ we sought to determine whether and how this is MPTP dependent. We found CCK-8-induced trypsin activity significantly inhibited in *Ppif^−/−^* compared with Wt (figure 5A), despite no differences in the amount of trypsinogen (or amylase) between Wt and *Ppif^−/−^* mice pancreata (figure 5B; nor cathepsin B, Bcl-xL or Bcl-2, data not shown). This finding indicates that MPTP opening contributes to pathological, intra-acinar zymogen activation. Zymogen activation depends on intracellular calcium overload^30^ and accumulation of activated zymogens in AP is due to impaired autophagy.^41^ We therefore measured levels of microtubule-associated protein 1A/1B-light chain 3 (LC3), which in autophagy is converted from cytosolic LC3-I to lipidated LC3-II and recruited into autophagosomal membranes, and levels of sequestosome 1 (SQSTM1, p62), which sequesters ubiquitinated protein aggregates to autophagosomes; when autophagosomes fuse with lysosomes, both LC3-II and p62 are degraded.^42^ Following induction of CER-AP that features marked falls in ATP production, acinar cell vacuolisation and zymogen activation,^7^ ^38^ ^43^ significant increases in LC3-II and p62 occurred in Wt pancreata, showing retarded autophagy consistent with previous data.^41^ Increases in LC3-II and p62 were significantly attenuated in *Ppif^−/−^* mice (figure 5C–E), indicating more efficient autophagy.^42^ We confirmed the role of MPTP opening in defective autophagy using GFP LC3 mice,^21^ crossed with *Ppif^−/−^* mice. Analysis of LC3 puncta (autophagic vacuoles, figure 5F) as well as increases in LC3-II and p62 in GFP-LC3 versus GFP-LC3×*Ppif^−/−^* mice (≥3 mice/group, data not shown) confirmed significant attenuation from genetic inhibition of the MPTP.

### Genetic or pharmacological MPTP inhibition sustains ATP production and confers striking protection from experimental AP

**Figure 6:**
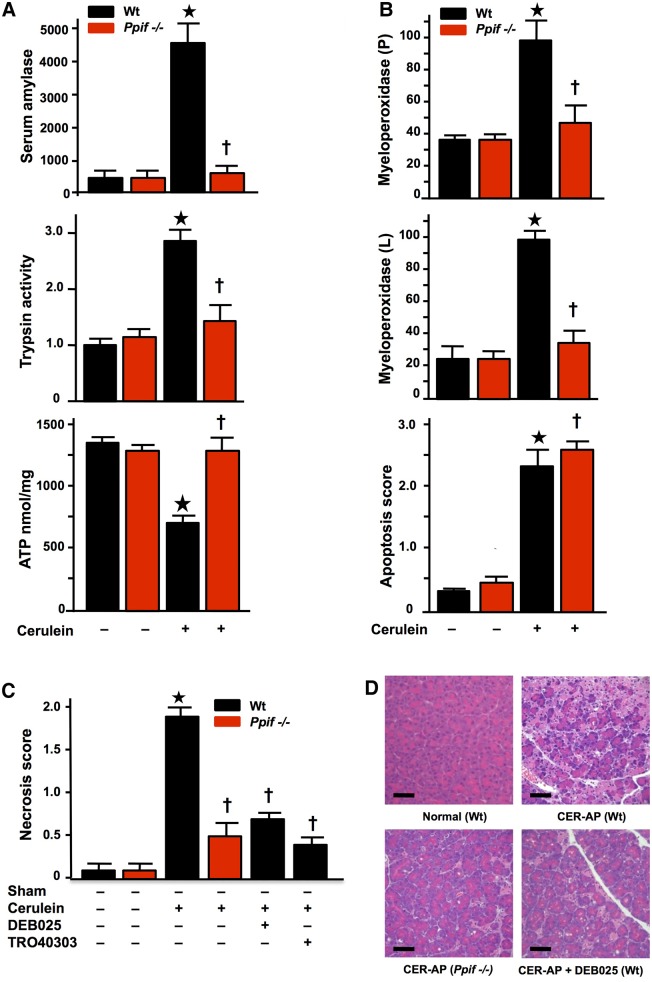
Genetic and pharmacological mitochondrial permeability transition pore (MPTP) inhibition markedly reduces the severity of caerulein acute pancreatitis (CER-AP). (A) CER-AP resulted in substantial elevations of serum amylase (U/L) and pancreatic trypsin (normalised to wild type (Wt) saline controls) with substantial reduction in pancreatic ATP content in Wt (*p<0.05) but not *Ppif^−/−^* mice (^†^p<0.05 vs CER-AP in Wt). (B) CER-AP resulted in substantial elevations of pancreatic (P) and lung (L) myeloperoxidase activity (normalised to CER-AP in Wt at 100) in Wt (*p<0.05) but not *Ppif^−/−^* mice (^†^p<0.05 vs CER-AP in Wt), while apoptosis scores were significantly increased in CER-AP in both Wt (*p<0.05 vs either control) and *Ppif^−/−^* (^†^p<0.05 vs either control). (C) Necrosis scores in CER-AP were substantially reduced in *Ppif^−/−^* and Wt with DEB025 (10 mg/kg intraperitoneal with third injection of caerulein) or TRO40303 (3 mg/kg intraperitoneal at same time points) compared to Wt with no treatment (all values means±SEM from ≥6 mice per group in all experiments; *p<0.01 CER-AP in Wt vs Wt controls; ^†^p<0.05 CER-AP in *Ppif^−/−^* or Wt with DEB025 or TRO40303 vs CER-AP in Wt). (D) Normal pancreatic histology (Wt no treatment) contrasted with CER-AP in Wt, *Ppif^−/−^* or Wt treated with DEB025, showing extensive oedema, necrosis and inflammatory cell infiltration in Wt but not *Ppif^−/−^* and not in Wt with DEB025 (H&E, black bars=50 μm).

**Figure 7:**
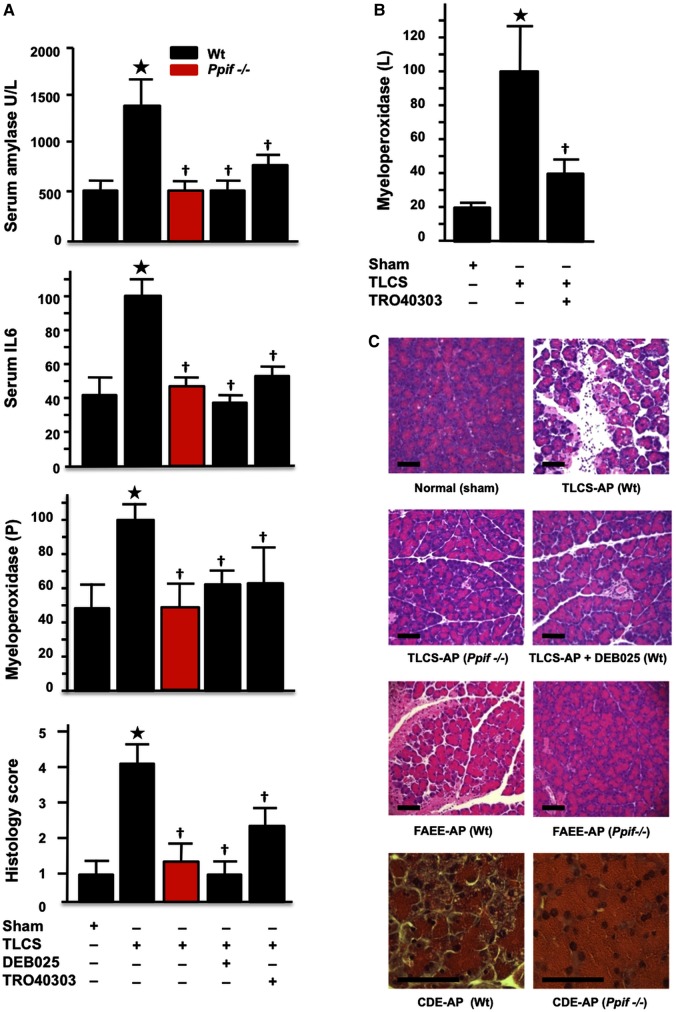
Genetic or pharmacological mitochondrial permeability transition pore (MPTP) inhibition abolishes or markedly attenuates biochemical and histological responses of taurolithocholic acid sulfate acute pancreatitis (TLCS-AP), fatty acid ethyl ester (FAEE)-AP and choline-deficient ethionine-supplemented (CDE)-AP. (A) Characteristic elevations in TLCS-AP of serum amylase (U/L), interleukin-6 (pg/mL), pancreatic (P) myeloperoxidase activity (normalised to TLCS-AP in wild type (Wt) at 100) and histology scores (*p<0.05 for all elevations vs sham controls) were all significantly reduced in *Ppif^−/−^* or in Wt treated with DEB025 or TRO40303 (^†^p<0.05 vs TLCS-AP in Wt without treatment). (B) Characteristic elevations in lung (L) myeloperoxidase activity (normalised to TLCS-AP in Wt at 100; *p<0.05 vs sham controls) were significantly reduced in Wt treated with TRO40303 (^†^p<0.05 vs TLCS-AP in Wt without treatment). (E) Representative histology showing protective effects of *Ppif^−/−^* in TLCS-AP, of DEB025 on TLCS-AP in Wt and of *Ppif^−/−^* in FAEE-AP and CDE-AP.

To determine comprehensively the significance of these mechanisms in vivo, we compared responses of *Ppif^−/−^* versus Wt mice in four dissimilar models of AP: CER-AP, TLCS pancreatic ductal infusion^27^ (TLCS-AP), ethanol with POA^11^ (FAEE-AP) and CDE-AP diet.^28^ These models represent the whole spectrum of human AP, including the commonest clinical aetiologies (gallstones and ethanol) and extending from mild to lethal disease. In all models, characteristic changes occurred in serum amylase and interleukin-6 (IL-6), pancreatic trypsin and myeloperoxidase, pancreatic ATP and histopathology (figures 6 and 7, see online supplementary figures S3 and S4). In contrast, all pathological responses were greatly inhibited in *Ppif^−/−^* animals, including lung myeloperoxidase and IL-6, which mediates lung injury and lethality.^44^ In Wt, DEB025 (10 mg/kg) or TRO40303 (3 mg/kg) administered 2 h after the start of hyperstimulation in CER-AP (figure 6C, D, see online supplementary figure S3) or 1 h after induction of TLCS-AP (figure 7) markedly reduced or abolished all pathological changes. Protection in TLCS-AP was close to complete: all changes in *Ppif^−/−^* mice or Wt (C57BL/6) mice treated with DEB025 or TRO40303 were no or minimally different from sham controls (figure 7). These findings demonstrate that inhibition of MPTP opening confers striking local and systemic protection from pancreatitis. The further new finding of relative independence of apoptotic processes from the MPTP (figure 6B) confirmed that apoptosis is not a major contributor to the pathological responses of AP,^26^ unless it is massive.^45^

## Discussion

**Figure 8:**
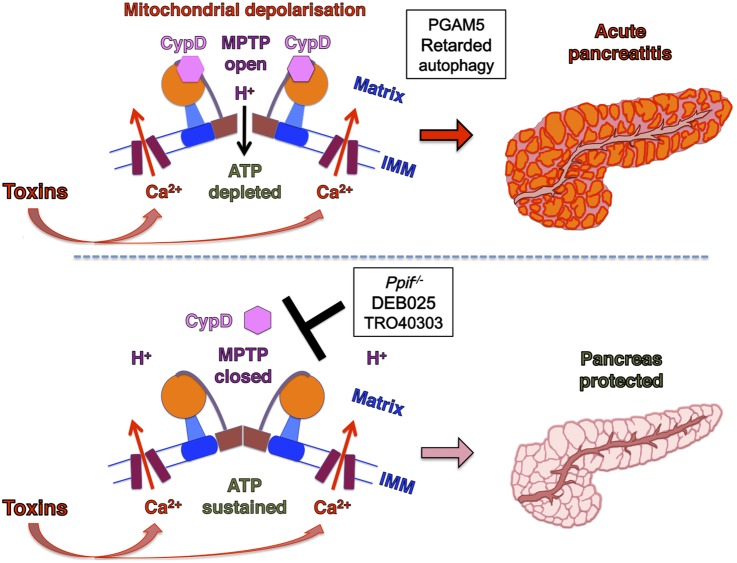
Summary diagram: the mitochondrial permeability transition pore (MPTP) plays a critical role in the development of acute pancreatitis. Exposure to pancreatic toxins leads to a sustained rise in cytoplasmic calcium that crosses the inner mitochondrial membrane (IMM) to enter the mitochondrial matrix. Consequent cyclophilin D (CypD) activation promotes MPTP opening across the IMM, causing mitochondrial depolarisation and impaired ATP production. These induce PGAM5 activation and retarded autophagy, downstream mechanisms in acute pancreatitis (upper panel). When MPTP opening is inhibited by genetic (Ppif^−/−^) or pharmacological means (DEB025 or TR040303), mitochondrial membrane potential is preserved and ATP production sustained. This maintains the integrity of pancreatic acinar cells that clear calcium more effectively and prevents the development of acute pancreatitis (lower panel) (MPTP drawn after reference 14).

This study demonstrates that MPTP opening is critical to experimental AP, mediating impaired ATP production, defective autophagy, zymogen activation, inflammatory responses and necrosis (figure 8), features of AP at molecular, cellular and whole organism levels.^1^ Our previous work identified metabolic effects of MPTP opening specific to ethanol. Here we have established the general significance of MPTP opening as a central mechanism in the pathogenesis of AP, and the primary role of calcium overload in this. The patch clamp data show how tight control of cytosolic calcium elevations essential to normal stimulus-secretion coupling by IP_3_Rs and RyRs^4^ is lost in Wt but maintained in *Ppif^−/−^* pancreatic acinar cells, which preserve ATP supply and clear calcium more effectively. Coupling of endoplasmic reticulum IP_3_Rs and RyRs with outer mitochondrial membranes tightly localises high calcium concentrations,^46^ but may expose mitochondria to abnormal calcium release, despite modulation by Bcl-2 family proteins.^7^ Here we have shown that pancreatitis toxins cause abnormal release of calcium via IP_3_Rs and RyRs that overloads pancreatic acinar mitochondria, which are markedly sensitive to calcium signals.^23^ The mitochondrial calcium overload induces high conductance MPTP opening and dissipates Δψ_m_, initiating collapse of ATP production, diminished calcium clearance, PGAM5 activation and subsequent necrosis. Importantly for a disease without specific treatment, pharmacological MPTP inhibition^29^ ^47^ administered after AP induction came close to preventing all injury, notably in the clinically relevant TLCS-AP.

For more than a century following an original postulate by Chiari,^48^ pancreatitis has been viewed as an autodigestive disease consequent on pathological zymogen activation.^3^ ^34^ ^38^ ^39^ ^45^ In experimental AP, zymogens are activated inside acinar cells within minutes of toxin exposure,^1^ ^3^ ^30^ ^41^ which this work has shown to result from induction of the MPTP, caused by and contributing to calcium overload. Sustained calcium overload may activate degradative calpains, phospholipases or other enzymes^17^ and damage zymogen granules, inducing autophagic^41^ and/or endolysosomal^49^ responses that activate digestive enzymes. Such activation was not completely prevented by MPTP inhibition, however, likely from global cytosolic calcium overload that was seen to be more effectively cleared in *Ppif^−/−^* cells, without which overload no enzyme activation occurs.^30^ Nevertheless, intracellular expression of trypsin per se without mitochondrial injury leads to apoptotic not necrotic pathway activation^45^ and trypsinogen activation does not appear necessary for either local or systemic inflammation;^50^ knockout of cathepsin B greatly reduces trypsinogen activation with little effect on serum IL-6 or lung injury.^39^ Hereditary pancreatitis caused by cationic trypsinogen gene mutations rarely features clinically significant pancreatic necrosis;^51^ ^52^ further, systemic protease inhibition has had little success as a clinical strategy,^1^ suggesting that while zymogen activation contributes, it is not the critical driver of AP. This study, however, shows that MPTP opening triggers defective autophagy, while inhibition of MPTP opening preserved ATP supply, increased the efficiency of autophagy and decreased zymogen activation. Together with major effects of MPTP opening on PGAM5 activation that implements necrosis,^19^ ^37^ and on local and systemic inflammatory responses, these findings now place mitochondrial injury centrally in AP.

Our new data show that in pancreatic acinar cells IP_3_Rs and RyRs are vulnerable to specific toxins that markedly increase their calcium channel open-state probabilities. Toxic transformation of calcium channel function induced pancreatic acinar cell necrosis through calcium-dependent formation of the MPTP, with diminished ATP production the critical consequence. Toxic transformation by different toxins was specific to different second messengers, identifying potential for a variety of deleterious effects. ATP deficiency may be further exacerbated by fatty acids released on hydrolysis of FAEEs or triglycerides,^53^ which may inhibit beta oxidation.^6^ Without sufficient ATP, cytosolic calcium overload produces a vicious circle in which high-affinity, low-capacity sarcoendoplasmic reticulum calcium transport ATPase (SERCA) and plasma membrane calcium ATPase (PMCA) pump clearance of cytosolic calcium is impaired, further mitochondrial injury sustained and necrotic cell death accelerated.^6^ ^12^ Although the toxicity of cytosolic calcium overload depends on calcium store refilling from outside the cell,^30^ ^54^ specific second messenger receptor blockade demonstrated calcium overload to be due completely to release from their calcium channels, not direct effects of toxins on calcium entry or extrusion.

Whereas the vast majority of previous studies undertaken to determine mechanisms and/or new targets in AP have used only one model, our four models are broadly representative of a range of aetiologies, including biliary (TLCS-AP), hyperstimulation (CER-AP), ethanolic (FAEE-AP) and amino acid-induced (CDE-AP).^1^ ^55^ Our findings in experimental AP are entirely consistent with those made in isolated mitochondria and cells, identifying a generalised mechanism of pancreatic injury and necrosis, confirmed in murine and human pancreatic acinar cells, pancreas lobules and tissue slices. Pancreatic necrosis drives the inflammasome,^56^ which can be induced by MPTP opening^57^ and is part of the systemic inflammatory response contributing to multiple organ failure.^2^ Further pancreatic injury is driven through tumour necrosis factor receptor activation that also promotes MPTP opening^58^ and calcium deregulation, activating calcineurin and NFAT.^59^ Our data link necrosis and inflammation directly, highlighting the potential of the MPTP as a drug target for AP.

## Supplementary Material

Web supplement

Web abbrevations

Web figures

Web table
