# Correction: *Mechanism of mitochondrial permeability transition pore induction and damage in the pancreas: inhibition prevents acute pancreatitis by protecting production of ATP*

**DOI:** 10.1136/gutjnl-2014-308553corr1

**Published:** 2019-04-03

Mukherjee R, Mareninova OA, Odinokova, *et al*. Mechanism of mitochondrial permeability transition pore induction and damage in the pancreas: inhibition prevents acute pancreatitis by protecting production of ATP. *Gut* 2016;65:1333-46. doi: 10.1136/gutjnl-2014-308553.

The authors wish to correct the collaborator statement in the paper. It should read as follows:

Collaborators: The authors are indebted to members of the NIHR Pancreas BRU: Diane Latawiec for technical assistance, Dayani Rajamanoharan for pancreatic lobule assays, Euan Mclaughlin for whole cell assays with caffeine and Paula Ghaneh, Christopher Halloran and Michael GT Raraty for provision of human pancreas tissue samples. The authors wish to thank Dr Samuel W French (Harbor-UCLA Medical Center, Torrance, CA) for providing electron micrographs that show mitochondrial damage in CER-AP.

