# Supplementary material for: Mechanism of mitochondrial permeability transition pore induction and damage in the pancreas: inhibition prevents acute pancreatitis by protecting production of ATP
Source: Gut. 2015 Jun 12;65(8):1333–46. doi: 10.1136/gutjnl-2014-308553 (PMC4920725; doi:10.1136/gutjnl-2014-308553)
Supplement: Web supplement [file gutjnl-2014-308553-s1.pdf]

Supplementary Methods

Pancreas tissue slices and lobules. Human pancreas tissue was set in 10% gelatine and sliced with a Leica VT1200 Vibratome (Leica Microsystems Gmbh) at ~150 μm thickness. Slices were loaded with TMRM (10 μM, 20 min, dequench mode[23]), washed, and measured using a PolarStar Omega BMG Labtech microplate reader (excitation 540 nm, emission 590 nm). Murine pancreas lobules were dissociated manually and placed in (mM): 140 NaCl, 4.7 KCl, 1.13 $MgCl_2$, 1 $CaCl_2$, 10 D-glucose, 10 HEPES (adjusted to pH 7.35 using NaOH).

Isolated mitochondria responses. Measurements were made on mitochondria in *medium A* containing 250 mM sucrose, 22 mM KCl, 22 mM triethanolamine (pH 7.4), 3 mM $MgCl_2$, 5 mM KH_2PO_4; or *medium B* containing 100 mM KCl, 30 mM 3-morpholinopropanesulfonic acid (pH 7.2), 3 mM $MgCl_2$, 5 mM KH_2PO_4, and 10 mM calcium/EGTA buffers; 10 mM succinate was used as the respiratory substrate. Oxygen consumption was measured using a Clark-type electrode (Instech Lab) and oxygen meter (Yellow Springs Instruments). Membrane potential was monitored in presence of 1 μM tetraphenyl phosphonium (TPP^+) with TPP^+-sensitive electrode[9,26].

Electron microscopy. Pancreatic tissue was cut into 1 mm cubes and fixed overnight at 4°C in 2.5% glutaraldehyde, 0.15 M sodium cacodylate (pH 7.4). After post-fixation in 1% OsO_4 followed by uranyl acetate, tissue was dehydrated in ethanol and embedded in epoxy resin. 100 nm thick sections were examined in a Hitachi 600 electron microscope.

Immunofluorescence. For immunolabeling, murine pancreata were dissected and fixed as described[37]. Images were acquired with a Zeiss LSM

710 confocal microscope using x 63 objective. Nuclei were counterstained with DAPI.

Enzyme activity, ATP and IL-6 determination. Trypsin activity was measured in homogenized cells/tissue, using Boc-Gln-Ala-Arg-AMC substrate converted by trypsin to a fluorescent product (excitation 380 nm, emission 440 nm) [37]. Serum amylase was measured by Hitachi 707 (Antech Diagnostics) or Roche Analyzer (Roche), and serum IL-6 levels by Quantikine ELISA (R&D Systems). ATP levels were measured in pancreatic homogenates by luciferin/luciferase-based ATP determination kit (Molecular Probes)[7], with TD 20/20 luminometer (Turner Designs).

Myeloperoxidase assay. Myeloperoxidase activity was measured by a bromide-dependent fluorometric assay[41]. Pancreatic tissue was homogenized, resuspended in 100 mM potassium phosphate buffer (pH 5.4) containing 0.5% hexadecyltrimethyl ammonium bromide, 10 mM EDTA and protease inhibitors, freeze-thawed three times, sonicated for 30 sec and centrifuged for 15 min at 16,000 × g. Myeloperoxidase activity was measured in supernatants mixed with 3,3,5,5-tetramethylbenzidine as substrate (1.6 mM, final) with freshly added H_2O_2 (3 mM, final; absorbance at 655 nm for 3 min at 30 sec intervals). Activity was calculated by standard curve and expressed as units/mg protein or normalized to control.

Immunoblot analysis. Immunoblotting of pancreatic tissue, isolated mitochondrial homogenates, membrane or cytosolic fractions was as described[44]. Protein concentration was determined by Bradford assay (Bio-Rad Laboratories). Blots were visualized by enhanced chemiluminescence detection kit (Pierce). Band intensities were quantified by densitometry using

Scion imaging software (Scion Corporation) or FluorChem HD2 imaging system (Alpha Innotech). Cytochrome c release was measured by immunoblot[44].

Chemicals and reagents. CCK-8 was from American Peptide; caerulein, Peninsula Laboratories; Boc-Gln-Ala-Arg-AMC, Peptides International; TMRM, Invitrogen other fluorescent dyes, Molecular Probes; antibody against cytochrome c, BD Biosciences; anti-COX IV, Molecular Probes; anti-cyclophilin D, Affinity Bioreagents; anti-trypsin, Chemicon International; anti-PGAM5, Santa Cruz Biotechnology Inc; anti-LC3B and anti-SQSTM1/p62, Cell Signaling Technology. Purified trypsin was from Worthington; calcium/EGTA buffer kit; Molecular Probes; IL-6 ELISA kit, R&D Systems Europe Ltd; other reagents, Sigma Chemical; D-MeAla^{3}-EtVal^{4}-cyclosporin (Alisporivir, DEB025), Debiopharm S.A.; 3,5-Seco-4-nor-cholestan-5-one oxime-3-ol (TRO40303), Trophos S.A..
