# Supplementary material for: Mechanism of mitochondrial permeability transition pore induction and damage in the pancreas: inhibition prevents acute pancreatitis by protecting production of ATP
Source: Gut. 2015 Jun 12;65(8):1333–46. doi: 10.1136/gutjnl-2014-308553 (PMC4920725; doi:10.1136/gutjnl-2014-308553)
Supplement: Web abbrevations [file gutjnl-2014-308553-s2.pdf]

List of Abbreviations:

ACh:	acetylcholine
ADP:	adenosine diphosphate
AP:	acute pancreatitis
ATP:	adenosine triphosphate
BAPTA:	1,2-bis(o-aminophenoxy)ethane-N,N,N',N'-tetraacetic acid
Bcl2:	B-cell lymphoma 2
BKA:	bongkrekic acid
CCCP:	carbonyl cyanide *m*-chlorophenyl hydrazone
CCK:	cholecystokinin
CDE:	choline-deficient ethionine-supplemented
CER:	caerulein
CYA:	cyclosporin A
CypD:	cyclophilin D
DCFDA:	dichlorodihydrofluorescein diacetate acetyl ester
DEB025:	alisporivir
EGTA:	ethylene glycol tetraacetic acid
ELISA:	enzyme-linked immunosorbent assay
ERK:	extracellular-signal regulated kinase
FAEE:	fatty acid ethyl ester
GFP:	green fluorescent protein
H&E:	haematoxylin and eosin
ICl_{Ca}:	calcium-activated Cl^- currents
IMM:	inner mitochondrial membrane
IP_3:	inositol trisphosphate
IP_3R:	inositol trisphosphate receptor
LC3:	microtubule-associated protein 1A/1B-light chain 3
MEN:	menadione
MPTP:	mitochondrial permeability transition pore
NAADP:	nicotinic acid adenine dinucleotide phosphate
NAD(P)H:	nicotinamide adenine dinucleotide (phosphate)
NFAT:	nuclear factor of activated T-cells
PGAM5:	phosphoglycerate mutase family member 5
PI:	propidium iodide
POA:	palmitoleic acid
POAEE:	palmitoleic acid ethyl ester
PPI:	peptidyl-prolyl isomerase
Ppif:	peptidyl-prolyl isomerase F (also known as D) gene
R110:	rhodamine 110
ROS:	reactive oxygen species
RyR:	ryanodine receptor
SO:	sytox orange
SQSTM1:	sequestosome 1 (also known as p62)
TLCS:	taurolithocholic acid sulphate
TMRM:	tetramethyl rhodamine methyl ester
TPP:	tetraphenyl phosphonium
TRO40303:	3,5-Seco-4-nor-cholestan-5-one oxime-3-ol
TUNEL:	terminal deoxynucleotidyl transferase dUTP nick end labeling
Wt:	wild type
