# Supplementary material for: Mechanism of mitochondrial permeability transition pore induction and damage in the pancreas: inhibition prevents acute pancreatitis by protecting production of ATP
Source: Gut. 2015 Jun 12;65(8):1333–46. doi: 10.1136/gutjnl-2014-308553 (PMC4920725; doi:10.1136/gutjnl-2014-308553)
Supplement: Web figures [file gutjnl-2014-308553-s3.pdf]

Figure S1

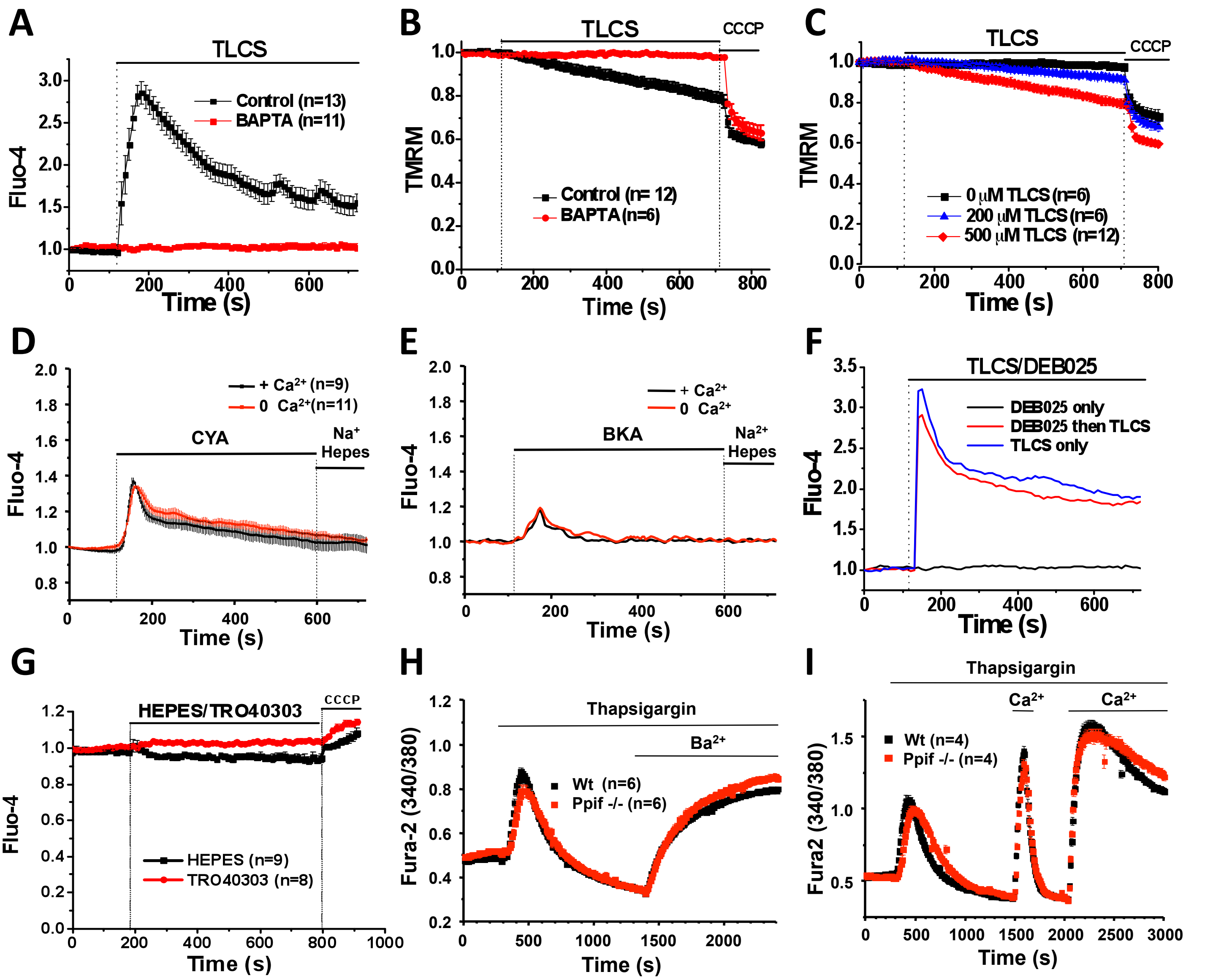

Figure S1. Effects of TLCS and pharmacological or genetic MPTP inhibition on cytosolic calcium (Fluo-4 or Fura-2) and mitochondrial membrane potential (TMRM) responses of murine pancreatic acinar cells (mean ± s.e.m. ratio to basal, F/F_0; n = no. of experiments, each with many cells). (**A**) Effect of preloading with calcium chelator BAPTA on calcium and (**B**) $\Delta\psi_m$ responses to TLCS (500 μM) in comparison to cells without BAPTA. (**C**) Effect of TLCS on $\Delta\psi_m$ was dose dependent; the higher the concentration of TLCS, the greater the fall of $\Delta\psi_m$. (**D**) CYA-induced intracellular calcium release on first application of CYA, without (100 nM EGTA) or with extracellular calcium; (**E**) typical traces of BKA-induced calcium release without (100 nM EGTA) or with calcium. (**F**) DEB025 alone induced no cytosolic calcium change; DEB025 as pre-treatment followed by TLCS (labelled: DEB025 then TLCS) resulted in the same calcium changes as with TLCS only. (**G**) TRO40303 did not affect cytosolic calcium levels (CCCP protonophore control). (**H**) Calcium store depletion with thapsigargin (20 μM) in zero Ca^{2+} (100 nM EGTA) followed by extracellular barium (10 mM) entry (which is not extruded) showing similar entry rates in Wt and *Ppif*$^{-/-}$ cells confirmed in (**I**) with similar store depletion in zero Ca^{2+} followed by extracellular calcium (5 mM) entry, extrusion and re-entry in Wt and *Ppif*$^{-/-}$ cells (H and I mean ± s.e.m. ratio to basal, F/F_0).

Figure S2. Factors determining toxic globalisation of second messenger calcium release in response to pancreatitis toxins (*insets*, green Fluo-4 or red PI fluorescent cells, white bars = 10 µm). (**A**) *Top plot*: typical calcium spikes (Fluo-4, F/F_0, blue) elicited by patched IP_3 (1-10 µM) were transformed into global, prolonged elevations matched by ICl_{Ca} and non-specific cation currents upon POAEE (10 µM) application, inhibited by caffeine (Caf, pink); *middle plot*: sustained caffeine inhibition prevented toxic transformation and PI uptake from TLCS (10 µM) after IP_3; *bottom plot*: caffeine prevented toxic transformation and PI uptake from POAEE (10 µM) after IP_3. (**B**) Without external calcium, globalised signals (ICl_{Ca}) from POAEE after IP_3 decreased then disappeared, with no PI uptake. (**C**) TLCS (10 µM) induced toxic transformation of typical ICl_{Ca} elicited by cADPR (10 µM) not NAADP (100 nM) whereas POAEE (10 µM) induced toxic globalisation with NAADP (100 nM) not cADPR (10 µM); following transformation, early PI uptake prevented by patched ATP (red ICl_{Ca} with PI uptake, 0 mM ATP; black ICl_{Ca} no PI uptake, 4 mM ATP; two recordings superimposed in top and bottom plots). (**D**) *Top plot*: TLCS (10 µM) induced toxic globalisation of CCK-8-(1-5 pM) elicited calcium signals with PI uptake; *bottom plot*: control data showing no change in ATP (Mg Green) with patch, but decline after CCCP. (**E**) CCK-8 or ACh with TLCS (10 µM) or POAEE (10 µM) resulted in significantly increased PI uptake compared to TLCS (*$p<0.05$) or POAEE (†$p<0.05$) alone, without patch.

Figure S2

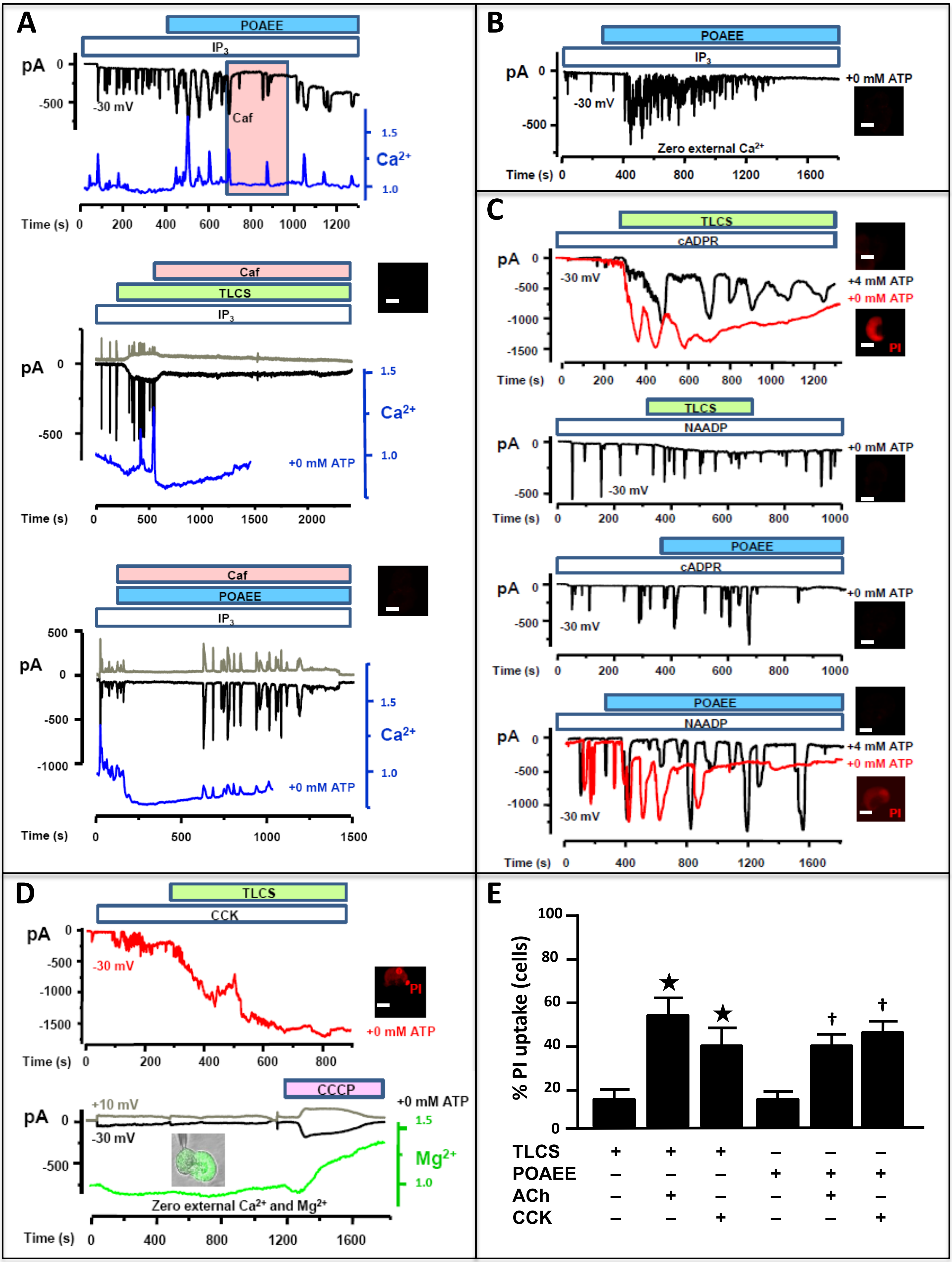

A
POAEE
IP3
pA 0
-500
-30 mV
Caf
1.5
Ca2+
1.0
Time (s) 0 200 400 600 800 1000 1200
Caf
TLCS
IP3
pA 0
-500
1.5
Ca2+
1.0
+0 mM ATP
Time (s) 0 500 1000 1500 2000
Caf
POAEE
IP3
500
pA 0
-500
-1000
1.5
Ca2+
1.0
+0 mM ATP
Time (s) 0 500 1000 1500
B
POAEE
IP3
pA 0
-500
-30 mV
+0 mM ATP
Zero external Ca2+
Time (s) 0 400 800 1200 1600
C
TLCS
cADPR
pA 0
-500
-1000
-1500
-30 mV
+4 mM ATP
+0 mM ATP
PI
Time (s) 0 200 400 600 800 1000 1200
TLCS
NAADP
pA 0
-500
-30 mV
+0 mM ATP
Time (s) 0 200 400 600 800 1000
POAEE
cADPR
pA 0
-500
-30 mV
+0 mM ATP
Time (s) 0 200 400 600 800 1000
POAEE
NAADP
pA 0
-500
-1000
-30 mV
+4 mM ATP
+0 mM ATP
PI
Time (s) 0 400 800 1200 1600
D
TLCS
CCK
pA 0
-500
-1000
-1500
-30 mV
PI
+0 mM ATP
Time (s) 0 200 400 600 800
CCCP
+0 mM ATP
pA 0
+10 mV
-30 mV
-500
1.5
Mg2+
1.0
Zero external Ca2+ and Mg2+
Time (s) 0 400 800 1200 1600
E
100
80
60
40
20
0
% PI uptake (cells)
TLCS + + + – – –
POAEE – – – + + +
ACh – + – – + –
CCK – – + – – +

Figure S3

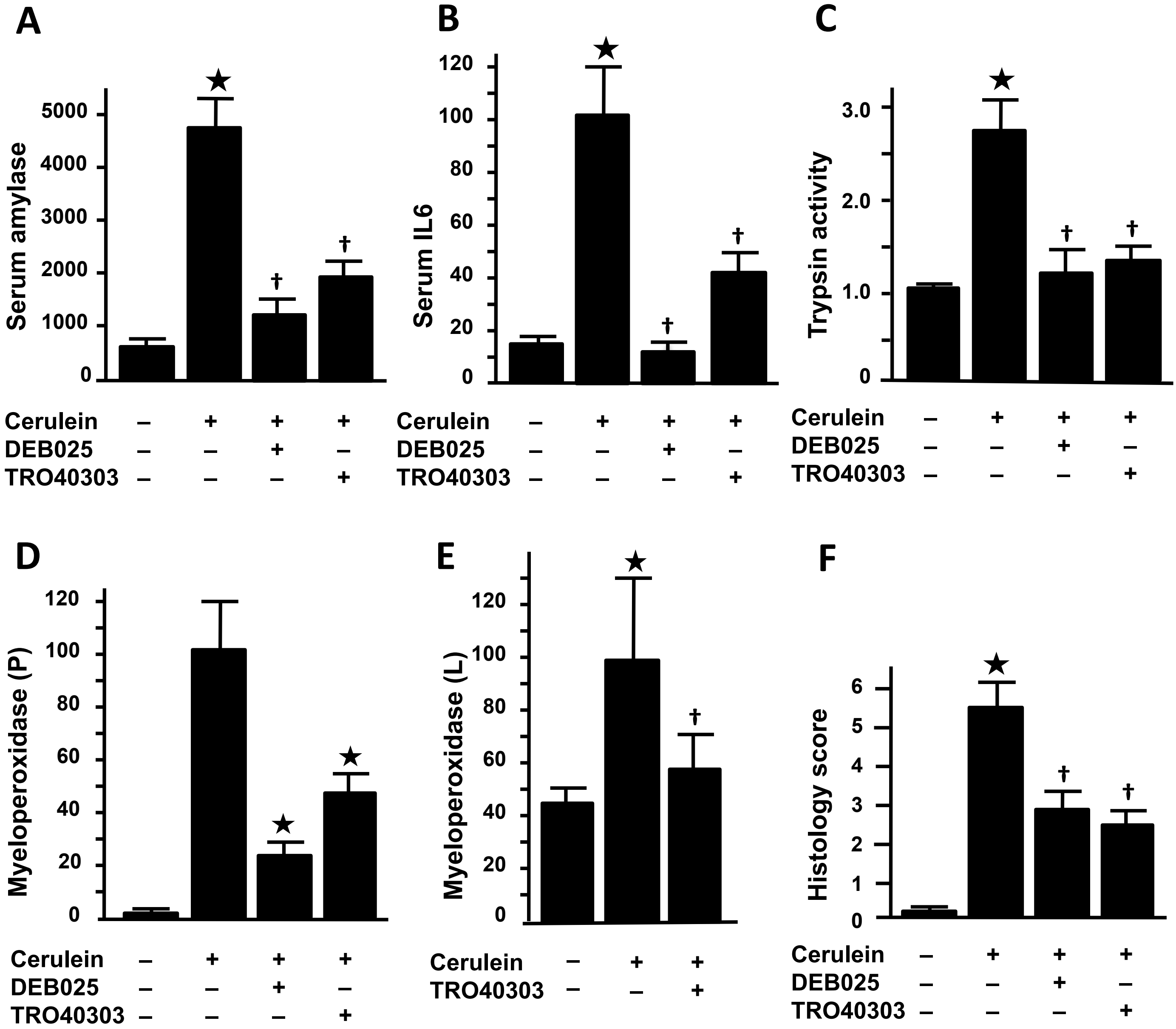

Figure S3. Pharmacological MPTP inhibition with DEB025 or TRO40303 markedly attenuates biochemical and histological responses of CER-AP in Wt mice. Characteristic elevations in CER-AP of (**A**) serum amylase (U/l) and (**B**) IL-6 (pg/ml), (**C**) pancreatic trypsin activity (normalized to saline controls at 1.0), (**D**) pancreatic (P) and (**E**) lung (L) myeloperoxidase activity (normalized to CER-AP at 100) and (**F**) histology scores (oedema, inflammatory infiltrate and necrosis) were all significantly reduced by treatment with DEB025 or TRO40303 (*p<0.05 for all elevations vs saline controls; †p<0.05 vs CER-AP without treatment).

Figure S4

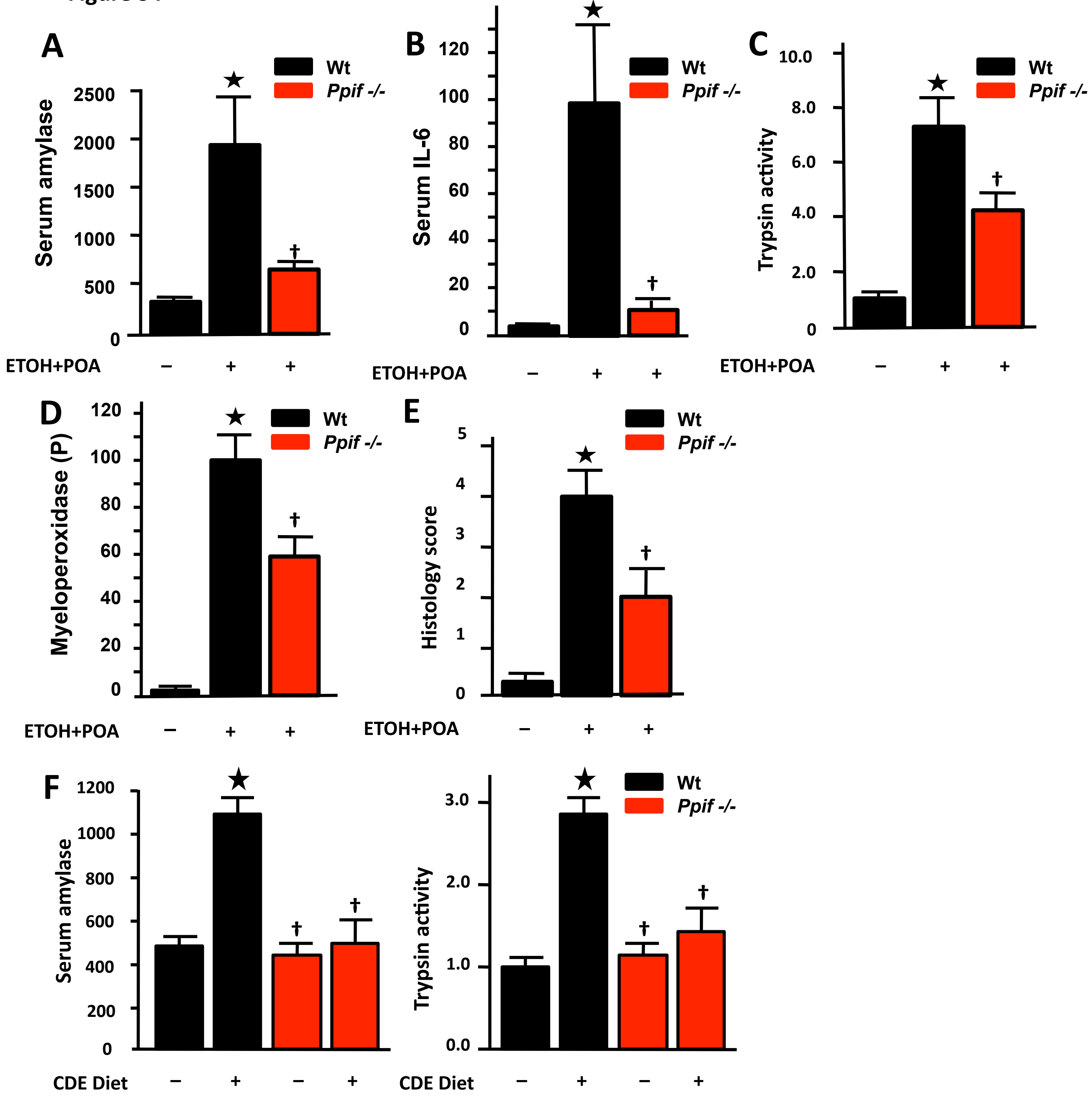

Figure S4. Genetic MPTP inhibition (*Ppif*$^{-/-}$) markedly attenuates biochemical and histological responses of FAEE-AP and CDE-AP. Characteristic elevations in FAEE-AP (Huang et al., 2013) of (**A**) serum amylase (U/l) and (**B**) IL-6 (pg/ml), (**C**) pancreatic trypsin activity (normalized to saline controls at 1.0), (**D**) pancreatic (P) myeloperoxidase activity (normalized to FAEE-AP at 100) and (**E**) histology scores (*p<0.05 vs Wt saline controls) were all significantly attenuated in *Ppif*$^{-/-}$ mice (†p<0.05 vs FAEE-AP in Wt). Characteristic elevations in CDE-AP of (**F**) serum amylase (*left*) and pancreatic trypsin activity (normalized to Wt controls receiving normal diet; *p<0.05 vs Wt controls, *right*) were abolished in *Ppif*$^{-/-}$ animals (†p<0.05 vs CDE-AP in Wt).
