# Supplementary material for: Mechanism of mitochondrial permeability transition pore induction and damage in the pancreas: inhibition prevents acute pancreatitis by protecting production of ATP
Source: Gut. 2015 Jun 12;65(8):1333–46. doi: 10.1136/gutjnl-2014-308553 (PMC4920725; doi:10.1136/gutjnl-2014-308553)
Supplement: Web table [file gutjnl-2014-308553-s4.pdf]

Table S1

Cells	Stimulus	Toxin	Caffeine	Pipette ATP	Globalisation[a]	PI uptake
Wt	1-10 μM IP_3	10 μM TLCS			6 of 6	5 of 6
Wt	1-10 μM IP_3	10 μM POAEE			9 of 9	8 of 9
Wt	1-10 μM IP_3	10 μM TLCS	20 mM		0 of 6	0 of 6[b]
Wt	1-10 μM IP_3	10 μM POAEE	20 mM		1 of 6	1 of 6[c]
Wt[d]	1-10 μM IP_3	10 μM TLCS			7 of 7[e]	1 of 7[c]
Wt[d]	1-10 μM IP_3	10 μM POAEE			7 of 7[e]	0 of 7[b]
Wt	1-10 μM IP_3	10 μM TLCS		4 mM	4 of 4[e]	0 of 4[c]
Wt	1-10 μM IP_3	10 μM POAEE		4 mM	4 of 4[e]	0 of 4[c]
Ppif$^{-/-}$	1-10 μM IP_3	10 μM TLCS			17 of 17[e]	0 of 17[b]
Wt	100nM NAADP	10 μM TLCS			1 of 13	N/A
Wt	100nM NAADP	10 μM POAEE			9 of 9	8 of 9
Wt	100nM NAADP	10 μM POAEE		4 mM	5 of 5[e]	0 of 5[b]
Wt	10 μM cADPR	10 μM TLCS			5 of 5	5 of 5
Wt	10 μM cADPR	10 μM TLCS		4 mM	4 of 4[e]	0 of 4[b]
Wt	10 μM cADPR	10 μM POAEE			0 of 12	N/A
Wt	1-5 pM CCK-8	10 μM TLCS			6 of 6	6 of 6
Wt	1-5 pM CCK-8	10 μM TLCS		4 mM	6 of 6[e]	0 of 6[b]
Wt	1-5 pM CCK-8	10 μM POAEE			7 of 7	7 of 7
Wt	1-5 pM CCK-8	10 μM POAEE		4 mM	5 of 5[e]	0 of 5[b]
Wt	20 nM ACh	10 μM TLCS			5 of 5	5 of 5
Wt	20 nM ACh	10 μM TLCS		4 mM	5 of 5[e]	0 of 5[b]
Wt	20 nM ACh	10 μM POAEE			6 of 6	6 of 6
Wt	20 nM ACh	10 μM POAEE		4 mM	6 of 6[e]	0 of 6[b]
Wt		200 μM TLCS			7 of 7	6 of 7
Wt		200 μM TLCS		4 mM	7 of 7[e]	0 of 7[b]
Ppif$^{-/-}$		200 μM TLCS			7 of 7[e]	0 of 7[b]

Table S1. Toxic globalisation of calcium signals and subsequent PI uptake elicited by quasi-physiological stimuli following application of pancreatitis toxins in Wt mouse pancreatic acinar cells, demonstrating protective effects of the IP_3R inhibitor caffeine or of no external calcium or of supplementary ATP in the pipette or of cyclophilin D knockout (*Ppif*$^{-/-}$; numbers of cells less than those given in main text as PI uptake was not tested in all cells from which ICl_{Ca} recordings were made).

[a]Globalisation characterised by transformation of apical into global calcium signals of >30 s duration.

[b]$p<0.01$ vs PI uptake in cells from respective Wt control group (i.e. calcium in external medium and no caffeine nor supplementary pipette ATP).

[c]$p<0.05$ vs PI uptake in cells from respective Wt control group (i.e. calcium in external medium with no caffeine nor supplementary pipette ATP).

[d]0 mM external calcium, demonstrating protection from PI uptake, as globalisation was not sustained in cells with external medium containing no calcium.

[e]Globalisation less marked/not sustained with no external calcium or with supplementary pipette ATP or in *Ppif*$^{-/-}$, showing return to baseline cytosolic calcium levels.
